# Antibacterial Activity of the Novel Drug Gepotidacin against *Stenotrophomonas maltophilia*—An In Vitro and In Vivo Study

**DOI:** 10.3390/antibiotics11020192

**Published:** 2022-02-01

**Authors:** Maike Isabell Sanders, Eyhab Ali, Jan Buer, Joerg Steinmann, Peter-Michael Rath, Hedda Luise Verhasselt, Lisa Kirchhoff

**Affiliations:** 1 Institute of Medical Microbiology, University Hospital Essen, University of Duisburg-Essen, 45122 Essen, Germany; maike.sanders@posteo.de (M.I.S.); eyhab21@hotmail.com (E.A.); jan.buer@uk-essen.de (J.B.); joerg.steinmann@klinikum-nuernberg.de (J.S.); peter-michael.rath@uk-essen.de (P.-M.R.); Hedda-luise.verhasselt@uk-essen.de (H.L.V.); 2 Institute of Clinical Hygiene, Medical Microbiology and Infectiology, Klinikum Nürnberg, Paracelsus Medical University, 90419 Nuremberg, Germany

**Keywords:** *Stenotrophomonas maltophilia*, gepotidacin, triazaacenaphthylene, topoisomerase inhibitor, *Galleria mellonella*, cystic fibrosis

## Abstract

*Stenotrophomonas maltophilia* is increasingly recognized as a nosocomial bacterial pathogen with a multi-drug resistance profile. In this study, the novel drug gepotidacin, the first compound of the novel triazaacenaphthylene topoisomerase inhibitor antibiotics class, was evaluated on its activity against clinical *S. maltophilia* isolates. Ninety-nine *S. maltophilia* isolates plus reference strain K279a (N = 100) were tested on their susceptibility towards gepotidacin in a broth microdilution. Additional susceptibility testing was performed towards the commonly applied combination trimethoprim/sulfamethoxazole (TMP/SXT), moxifloxacin, and levofloxacin. The time–kill kinetic of gepotidacin was observed in a time–kill assay. The greater wax moth *Galleria mellonella* was used to determine the activity of gepotidacin against *S. maltophilia* in vivo. Gepotidacin showed minimum inhibitory concentrations (MICs) between 0.25 and 16 mg/L (MIC_50_: 2 mg/L; MIC_90_: 8 mg/L), independently of its susceptibility towards TMP/SXT. The five TMP/SXT resistant strains exhibited gepotidacin MICs from 1 to 4 mg/L. The *S. maltophilia* strains resistant to the assessed fluoroquinolones showed in parts high MICs of gepotidacin. The time–kill assay revealed a time- and strain-dependent killing effect of gepotidacin. In vivo, injection of gepotidacin increased the survival rate of the larvae from 61 % to 90 % after 2 days. This study showed antimicrobial effects of gepotidacin towards *S. maltophilia*.

## 1. Introduction

*Stenotrophomonas maltophilia* is a non-fermentative, Gram-negative, opportunistic, ubiquitous bacterium. While it is known to occur in water and soil, it is also known to cause a broad range of infections in humans [1,2,3]. In particular, nosocomial infections with *S. maltophilia* are linked to a considerable mortality with a rate of 37.5 % [4]. The most frequently occurring infections with *S. maltophilia* are infections of the respiratory tract [5,6,7] and bacteremia [8,9].

Due to its elevated level of intrinsic resistances towards multiple classes of commonly applied antibiotics, e.g., carbapenems and aminoglycosides, and the description of acquired antimicrobial resistance mechanisms, e.g., against fluoroquinolones, and/or polymyxins, *S. maltophilia* is considered a human pathogen with a multi-drug resistance profile [1,10,11,12]. In addition to the reports on nosocomial infections in hospitalized and/or immunosuppressed patients, the chronic colonization of the respiratory tract of cystic fibrosis (CF) patients is also reported. With rates of ~10 %, colonization with *S. maltophilia* is an independent risk factor for pulmonary exacerbation of CF patients, associated with a decline in lung function and a worse outcome [13,14,15,16].

Today, the main therapeutic choice for infections with *S. maltophilia* is trimethoprim/sulfamethoxazole (co-trimoxazole, TMP/SXT) [17]. When treatment is not possible, e.g., due to resistances towards co-trimoxazole or patient’s intolerance, topoisomerase inhibitors such as ciprofloxacin or moxifloxacin as well as other antibiotics as ceftazidime and ticarcillin/clavulanate, alone or in combination with other antibiotics, may be considered for treatment [17]. However, the application of novel antibiotic classes in times of rising resistances, also towards fluoroquinolones, may become an important topic in the near future.

Gepotidacin (NBTI 5463; GSK2140944) is the first-in-class of the new bacterial topoisomerase inhibitors (NBTIs), known as triazaacenaphthylene bacterial type II topoisomerase inhibitor, showing activity against several bacterial species being resistant to fluoroquinolones [18,19,20]. In vitro data showed promising activity of gepotidacin against (methicillin-resistant) *Staphylococcus aureus, Streptococcus pneumoniae, Escherichia coli,* and *Neisseria gonorrhoeae* [21]. An ongoing clinical trial on single-dose treatment of urinary tract infection and uncomplicated urogenital gonorrhea with gepotidacin resulted in a 95 % efficiency against *N. gonorrhoeae* [22]. Gepotidacin inhibits the bacterial DNA-gyrase as well as the topoisomerase IV, using a distinct mechanism compared to the fluoroquinolones.

*S. maltophilia’s* multi-drug resistance together with increasing reports on nosocomial infections make *S. maltophilia* an emerging pathogen [23]. The aim of this study was to investigate the capability of gepotidacin to inhibit *S. maltophilia* growth in vitro. Additionally, the activity of gepotidacin against *S. maltophilia* infection was studied in vivo using the alternative infection model *Galleria mellonella*.

## 2. Results

**Figure 1:**
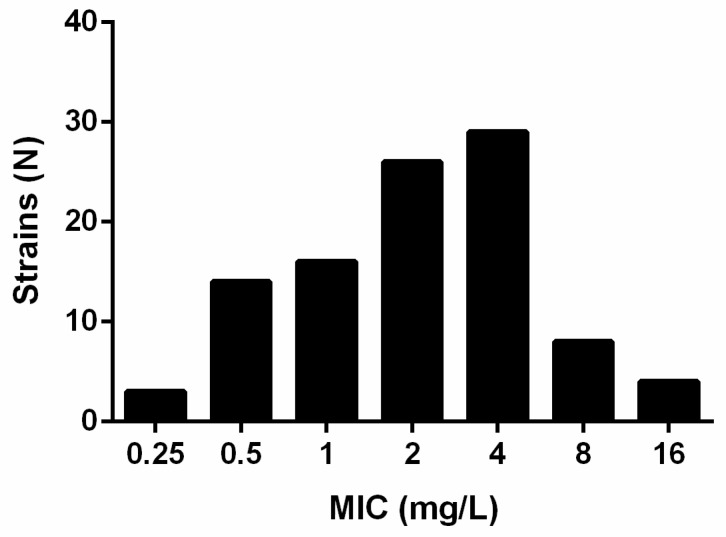
MIC (mg/L) distribution of N = 100 *Stenotrophomonas maltophilia* strains for gepotidacin as found in broth microdilution after EUCAST.

In vitro, gepotidacin showed a strain-dependent activity towards the here included isolates with minimum inhibitory concentrations (MICs) ranging from 0.25 to 16 mg/L (Figure 1).

The MIC_50_ and MIC_90_ were determined to be 2 and 8 mg/L, respectively. The MIC_90_ of the fluoroquinolones (N = 100) were 4 mg/L for levofloxacin and 2 mg/L for moxifloxacin (Table 1).

Susceptibility testing by MIC test strips revealed 15 (15 %) co-trimoxazole resistant strains. MICs ranged from 0.016 to >32 mg/L and the determined MIC_50_ and MIC_90_ were 0.19 and >32 mg/L, respectively.

**Figure 2:**
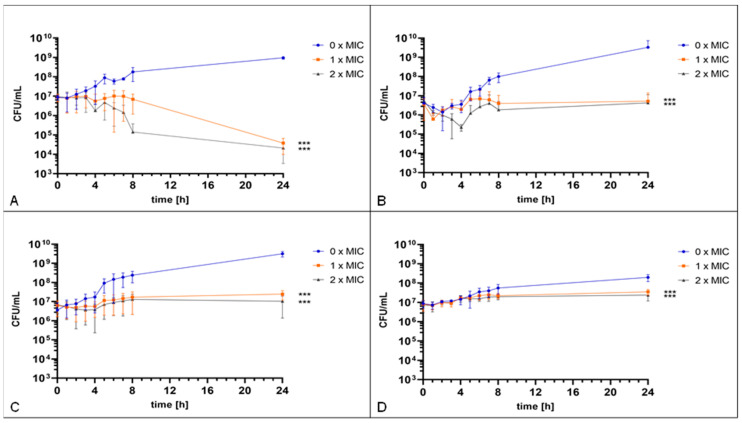
Time–kill assay. The reference strain K279a (**A**) and Sm 538 (**C**) were incubated with 0 mg/L, 4 mg/L, and 8 mg/L gepotidacin. The strain Sm 290 (**B**) was incubated with 0 mg/L, 1 mg/L, and 2 mg/L gepotidacin, while the strain Sm 1222 (**D**) was incubated with 0 mg/L, 2 mg/L, and 4 mg/L gepotidacin. CFU/mL were determined hourly over the first eight hours and after 24 h. Observed growth after incubation without gepotidacin (blue), with one-fold MIC (orange) and two-fold (grey) gepotidacin. Significance levels are shown by asterisks: ***: *p* < 0.001 and determined via two-way ANOVA using GraphPad Prism.

For further analysis, four strains (two wild type (K279a, Sm 538) and two co-trimoxazole resistant isolates (Sm 290, Sm 1222)) were included in a time–kill assay, determining the antimicrobial effect of gepotidacin against *S. maltophilia* over time. A significant growth inhibiting effect of two-fold of the MIC has been observed for each of the strains. However, the impact on viability of *S. maltophilia* varied between the different strains. For reference strain K279a, a significant reduction of ~4.5 log colony forming units (CFU)/mL was detected after 24 h of incubation compared to the non-treated control (Figure 2A). For two other strains, Sm 290 and Sm 538, after a reduction of 1.7 log and 2.5 log CFU/mL, respectively, compared to the non-treated control, a regrowth effect could be detected (Figure 2B,C). For the fourth strain, Sm 1222, characterized by slow growing behavior, the reduction in CFU was less with only 0.9 log CFU/mL after 24 h of incubation with gepotidacin when compared to the growth control (Figure 2D).

These in vitro results suggest an antimicrobial effect of gepotidacin against *S. maltophilia* in a strain-dependent manner.

**Figure 3:**
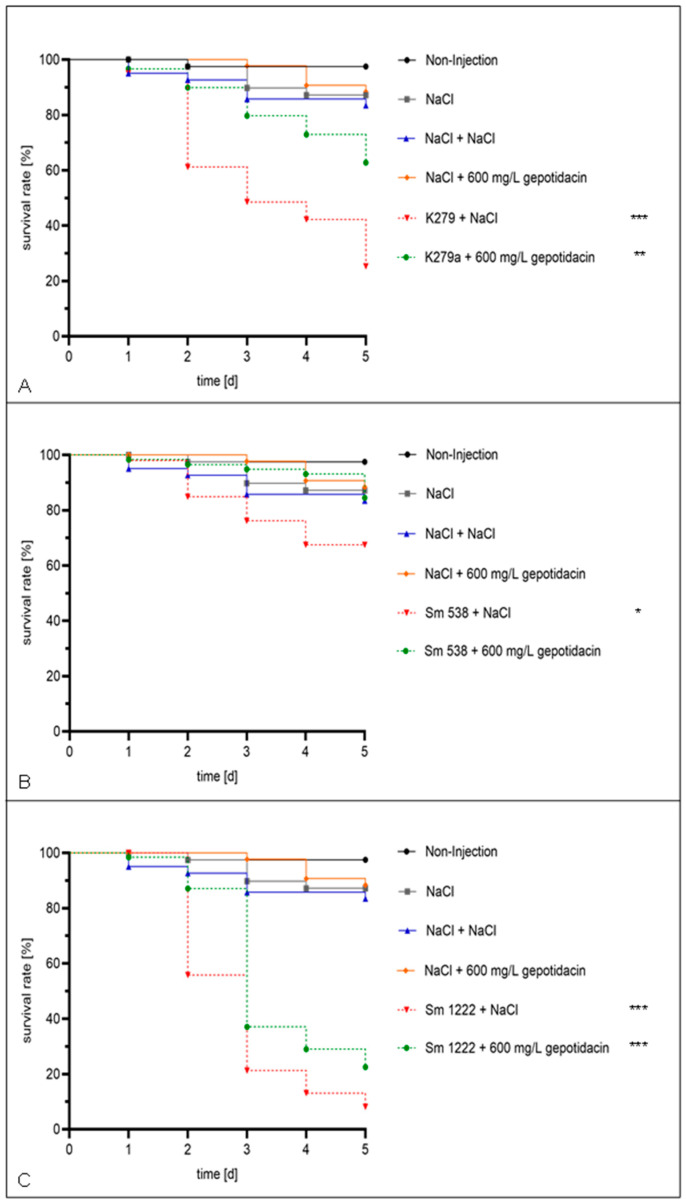
Single dose effect. The larvae were injected with a concentration of 10^5^ cells/mL and treated after 4 h with NaCl or 600 mg/L gepotidacin, respectively. Double-treatment controls with double injection of NaCl or gepotidacin were set up. The larvae were followed-up for five days. Showing the survival rate of the larvae, which were infected with K279a (**A**), Sm 538 (**B**), and Sm 1222 (**C**). Significance levels are shown by asterisks: *: *p* < 0.05, **: *p* < 0.005, ***: *p* < 0.001 and were determined via logrank test using GraphPad Prism.

A first evaluation of gepotidacin toxicity in *G. mellonella* revealed no death larvae when injected in concentrations of up to 32 mg/L. Additionally, the DMSO used for the solution of gepotidacin showed no toxicity in the tested concentrations of up to 10 % (data not shown). Virulence analysis revealed a strain specific virulence, with comparable high virulence of strain Sm 1222, moderate virulence of K279a and Sm 538, as well as low virulence of strain Sm 290. In the following, the latter was excluded from further in vivo antimicrobial activity testing with gepotidacin. Gepotidacin injection of the larvae four hours after infection with *S. maltophilia* in a concentration of 600 mg/L, equaling a dose of 20 mg/Kg body weight, resulted in an overall survival rate of 89.9 % after 2 d in comparison to the NaCl control group with 60.6 % (*p* = 0.0604). The effect of gepotidacin is strain dependent and more significant in the early phase of infection. After a monitoring period of 5 days, 53.1 % of the infected and treated larvae survived in comparison to the infected NaCl-control which resulted in 28.4 % survival (*p* = 0.073). Strain specific survival curves are depicted in Figure 3.

## 3. Discussion

We here demonstrated, for the first time, the antimicrobial activity of gepotidacin against *S. maltophilia* in an in vitro and in vivo study.

The here determined MICs of gepotidacin towards the tested *S. maltophilia* strains are comparable to those reported previously for Gram-negative bacteria with a MIC_50_ of 2 mg/L [21].

Comparing the MICs of gepotidacin against wild-type and co-trimoxazole resistant strains, higher MICs were found for those strains which were resistant towards co-trimoxazole. While, for co-trimoxazole wild-type strains, the MIC_50_ was 1 mg/L and the MIC_90_ 4 mg/L, the MIC_50_ and MIC_90_ of the co-trimoxazole resistant strains were higher with 2 and 16 mg/L, respectively. However, a correlation of these data could not be identified. Comparable results have been shown for strains resistant to at least one of the tested fluoroquinolones (MIC_90_: 16 mg/L), exhibiting higher MICs compared to fluroquinolone susceptible strains (MIC_90_: 2 mg/L). These findings are congruent to data from Biedenbach et al. showing distinct MICs of gepotidacin between levofloxacin-susceptible and resistant *E. coli* strains [24]. In another study, no association could be made between resistances towards different antibiotics including quinolones and increased MICs of gepotidacin [21].

While co-trimoxazole inhibits the synthesis of folic acid, fluoroquinolones target the bacterial topoisomerase II. Gepotidacin also affects the bacterial topoisomerases; thus, a link between co-trimoxazole resistance and high MICs of gepotidacin was not expected, whereas a link between elevated levofloxacin MICs and high gepotidacin MICs can be assumed.

Subsequently performed time–kill assays revealed an antibacterial activity of gepotidacin. Both, the MIC as well as the two-fold MIC showed a growth inhibiting effect over time on each of the tested strains. However, a strain-dependent killing with some strains showing a regrowth effect has been detected. Comparing strain K279a and strain Sm 538, both with similar MICs towards each of the tested agents, the antimicrobial effect of gepotidacin on K279a was stronger. A link between the susceptibility pattern and the strength of gepotidacin effect was thus excluded here. Strain-dependent differences in growth have also been reported for *N. gonorrhoeae* treated with gepotidacin in other studies [22].

The regrowth effect documented here for strain Sm 290 has also been described previously for *S. aureus* treated with gepotidacin, a decrease in viable cell count was followed by an increase, resulting in cell counts after 24 h like the concentration of time point at inoculation [21,25]. Lahiri et al. found a spontaneous mutation of the gyrase-subunit, enabling the regrowth of *S. aureus* after treatment with gepotidacin [25]. It is not to be excluded that a similar mutation might occur as well in some of the here assessed *S. maltophilia* isolates.

One of the here observed strains (Sm 1222) was characterized as slow growing. This strain revealed the least reduction in viable cell count compared to the other assessed strains with a reduction of 0.45 log CFU/mL after 24 h treatment with one-fold MIC (2 mg/L) in comparison to the non-treated control. It is suggested that the slow growth is indicating a less active metabolism which is responsible for the detected poor activity of gepotidacin in this time-kill assay. Each of the tested strains was not fully eradicated in the observed time period by giving the MIC or the two-fold MIC of gepotidacin.

We here demonstrated an activity of gepotidacin in vivo using the invertebrate infection model *G. mellonella*. A first test on virulence of the analyzed strains revealed a strain-dependent killing effect in the larvae. Strain Sm 1222 was characterized with a slow growing behavior, but virulence of this strain was high compared to the other strains, resulting in 0 % survival 6 days post inoculation. Gepotidacin showed no toxicity in *G. mellonella.* The tested concentration was previously safely examined in a rat pyelonephritis model [26]. Furthermore, Barth et al. assessed the safety and systemic exposure of gepotidacin in healthy adults as well as in adolescents. Their results promote an acceptable risk–safety profile in humans for gepotidacin [27].

In this in vivo model, larvae were infected with *S. maltophilia* and gepotidacin was injected 4 h post infection in a concentration equaling 20 mg/kg bodyweight. Larvae infected with each of the strains and subsequent injection of gepotidacin showed a significant higher survival compared to the control group. Even though *G. mellonella* is a suitable model for drug testing, further in vivo studies to determine gepotidacin activity against *S. maltophilia* should be performed, e.g., potentially in murine models.

## 4. Materials and Methods

### 4.1. Strains

All clinical samples were subject to conventional microbiological diagnosis before use. The study did not use demographic data about patients, nor did it result in any additional constraints for the patients. Because of the retrospective nature of the study, all data were anonymously analyzed without the need for patient consent. All procedures and methods were carried out in accordance with approved guidelines.

This study involved a total of 100 *S. maltophilia* strains; among them were 91 clinical and eight environmental strains plus the reference strain K279a. The clinical strains were isolated from respiratory specimens of CF patients (N = 70) and non-CF patients (N = 21). Species were identified by MALDI-TOF-MS (VITEK MS, bioMérieux, Nürtingen, Germany).

### 4.2. Susceptibility Testing

Susceptibility testing was performed for each included strain in a broth microdilution after EUCAST (European Committee on Antimicrobial Susceptibility Testing) guidelines, determining the MIC of gepotidacin towards each strain. Additionally, MICs of 100 strains towards levofloxacin and moxifloxacin were assessed in a broth microdilution. The anti-infective agents were diluted in solvent (dimethyl sulfoxide, DMSO) and Mueller Hinton Bouillon 2 for the preparation of stock solutions. Working solutions were prepared by dilution in Mueller Hinton Bouillon 2 and all solutions were stored at −20°C. Broth microdilution with gepotidacin, levofloxacin and moxifloxacin (MedChemExpress LLC, Monmouth, NJ, USA) was performed in concentrations between 0.03 and 16 mg/L. Additionally, MIC strip tests (Liofilchem srl, Roseto degli Abruzzi, Italy) were performed for co-trimoxazole for a total of 100 strains.

### 4.3. Time–Kill Assay

To determine the antimicrobial activity of gepotidacin in dependence to incubation time, a time–kill assay including four clinical strains, among them K279a, was performed. Viability of *S. maltophilia* was assessed hourly over 8 h and additionally checked after 24 h when incubated with gepotidacin in concentrations of previously identified MIC as well as the two-fold MIC. A respected growth control, not treated with gepotidacin, accompanied the tests. Incubation was performed in a microtiter plate and an inoculum of 1 × 10^7^ cells/mL in Mueller Hinton Broth 2 was prepared. Viability was determined by estimation of CFU/mL. Therefore, appropriate dilutions of the cell suspension were plated on Columbia Sheep Blood agar plates and incubated at 36 °C, until countable colonies were visible.

### 4.4. In Vivo Infection Assay

For the determination of gepotidacin activity against *S. maltophilia* infection in vivo, we infected the larvae of the greater wax moth *G. mellonella* with four different *S. maltophilia* strains. Infection was performed as with an inoculum of 1 × 10^5^ cells/mL in NaCl. In each run, each sample was injected into 15 larvae. As controls, a non-injection as well as a NaCl control was run additionally. After infection, larvae were incubated at 37 °C for 4 h before the animals were injected with 600 mg/L gepotidacin, equaling 20 mg/kg body weight according to the clinical trials [28]. Larvae were assessed daily on their survival. Dead larvae as well as pupae were noted respectively and removed from the experiment. Survival analysis after Kaplan–Meier was processed using GraphPad Prism8 (GraphPad Inc., La Jolla, CA, USA).

### 4.5. Statistics

All experiments were performed in triplicates. Statistical analysis was done in GraphPad Prism8. Significance levels are shown by asterisks: *: *p* < 0.05, **: *p* < 0.005, ***: *p* < 0.001. The applied statistical test is indicated in the corresponding figure legends.

## 5. Conclusions

In conclusion, we here demonstrated for the first time an antimicrobial activity of the novel drug gepotidacin against clinical *S. maltophilia* isolates in an in vitro and an in vivo study. In vitro, MICs varied between 0.25 and 16 mg/L. The time–kill assay revealed a time- and strain-dependent killing effect of gepotidacin. In vivo, injection of gepotidacin increased the survival rate of the larvae from 61 % to 90 % after 2 days.

## Figures and Tables

**Table 1:** Determined minimum inhibitory concentrations (MICs) of gepotidacin, levofloxacin, moxifloxacin, and co-trimoxazole against *S. maltophilia*.

Agent	N	Method	MIC (mg/L)		
			Range	MIC_50_	MIC_90_
Gepotidacin	100	Microdilution	0.25–16	2	8
Levofloxacin	100	Microdilution	0.25–>16	1	4
Moxifloxacin	100	Microdilution	≤0.003–>16	0.5	2
Co-trimoxazole	100	MIC test strips	0.016–>32	0.19	>32

## Data Availability

The data presented in this study are available on request from the corresponding author.
